# Pre- and Post-harvest Melatonin Application Boosted Phenolic Compounds Accumulation and Altered Respiratory Characters in Sweet Cherry Fruit

**DOI:** 10.3389/fnut.2021.695061

**Published:** 2021-06-09

**Authors:** Michail Michailidis, Georgia Tanou, Eirini Sarrou, Evangelos Karagiannis, Ioannis Ganopoulos, Stefan Martens, Athanassios Molassiotis

**Affiliations:** ^1^Laboratory of Pomology, Department of Horticulture, School of Agriculture, Aristotle University of Thessaloniki, Thessaloniki, Greece; ^2^Institute of Soil and Water Resources, Hellenic Agricultural Organisation (HAO-DEMETER), Thessaloniki, Greece; ^3^Institute of Plant Breeding and Genetic Resources, Hellenic Agricultural Organisation (HAO-DEMETER), Thessaloniki, Greece; ^4^Department of Food Quality and Nutrition, Centro Ricerca e Innovazione, Fondazione Edmund Mach, Trento, Italy

**Keywords:** melatonin, polyphenols, sweet cherry, pre-harvest, cold storage, anthocyanins, fruit ripening, respiration

## Abstract

The aim of the present study was to investigate the impact of exogenous melatonin (0. 5 mM) application through pre-harvest foliar spray and postharvest immersion, alone or in combination, on ripening parameters of sweet cherry (cv. Ferrovia) fruit and their relationship with bioactive compounds and gene expression at harvest as well after cold storage (0°C) for 12 days and subsequent room temperature (20°C) exposure for 8 h. Although several ripening traits were not influenced by melatonin, the combining pre- and post-harvest treatments delayed fruit softening at post-cold period. Preharvest spray with melatonin depressed fruit respiration at time of harvest while all applied treatments induced respiratory activity following cold, indicating that this anti-ripening action of melatonin is reversed by cold. Several genes related to the tricarboxylic acid cycle, such as *PaFUM, PaOGDH, PaIDH*, and *PaPDHA1* were upregulated in fruit exposed to melatonin, particularly following combined pre- and post-harvest application. The accumulation of phenolic compounds, such as neochlorogenic acid, chlorogenic acid, epicatechin, procyanidin B1, procyanidin B2+B4, cyanidin-3-*O*-galactoside, and cyanidin-3-*O*-rutinoside along with the expression of several genes involved in phenols biosynthesis, such as *PaSK, PaPAL, Pa4CL, PaC4H*, and *PaFNR* were at higher levels in melatonin-treated cherries at harvest and after cold exposure, the highest effects being observed in fruits subjected to both pre- and post-harvest treatments. This study provides a comprehensive understanding of melatonin-responsive ripening framework at different melatonin application conditions and sweet cherry stages, thereby helps to understand the action of this molecule in fruit physiology.

## Introduction

Sweet cherry (*Prunus avium* L.) is one of the popular and economically valuable fruit cultivated in temperate regions of the world and it is recognized for its nutraceutical properties (1). Due to their high respiratory activity, the minimal reserve carbohydrate, and the high susceptibility to mechanical damage, sweet cherries are highly perishable and have a shelf life of only about 2 weeks under cold chain management that includes rapid elimination of field heat after harvest and low temperature control during storage and shipping (2–5). Moreover, the physiological and biochemical mechanisms underlying fruit ripening following cold exposure are still relatively unknown for sweet cherries (6).

Melatonin (MT, *N*-acetyl-5-methoxytryptamine) is synthesized from tryptophan and is well-studied in mammals. Since the first discovery of melatonin in plants (7), a widespread existence in plant kingdom has been reported in a considerable variety of plant species. Melatonin plays an important role in many biological processes in plants, including seed germination, floral development, photosynthesis efficiency, maturation and senescence, osmotic adjustment and resistance to numerous environmental stresses (8). In addition to having plant growth regulator-like functions, a body of work has also revealed its role in fruit biology, such as depression of chilling injury symptoms, delay in ripening and decay incidence, and induced antioxidant and secondary metabolism (9–13). Recent studies have also highlighted the effect of exogenous melatonin on the post-harvest behavior of fruit, including sweet cherry (8). Nevertheless, an interpretation of the effects on combined pre- and post-harvest application on fruit metabolism during ripening and following cold exposure remain uncharacterized. Because an excess amount of melatonin could be harmful to post-harvest fruits (9), it also would be interesting to examine both positive and negative effects of melatonin on fruit ripening physiology.

Cherries have a relatively higher melatonin content than other fruits which makes them ideal for studying its function in fruits (14). The objective of the present study was to investigate the impact of melatonin applied by: (i) spraying before harvest, (ii) dipping after harvest, and (iii) the combination of pre- and post-harvest applications on sweet cherry ripening biology. To better understand the role of melatonin in sweet cherry ripening, as well as the effect of cold storage on melatonin function, we simultaneously analyzed various parameters associated with the ripening process at both harvest and post-cold period.

## Methods

### Treatments and Fruit Sampling

The experiment was conducted in a cherry orchard at the Farm of Aristotle University of Thessaloniki (Thermi, Thessaloniki). The orchard consisted of 11-years old sweet cherry (cv. Ferrovia) trees, planted at 5 × 5 m spacing between rows and along the row, grafted onto MaxMa 14 rootstock and in open vase training system. Application of melatonin [powder, ≥98% (TLC), Sigma-Aldrich, CAS Number 73-31-4] via foliar spray (0.5 mM) was performed in four trees, 2 and 1 weeks prior to harvest. Control trees were sprayed with water. Trees were subjected to standard horticultural practices and fruit ripening stage was defined according to the previous study (15). At harvest, fruit physiological traits and sampling of the two treatments, namely control and melatonin spray, were performed (marked as “harvest”). The cherries were selected for uniformity without any damage and randomly divided into two groups. Immediately after harvest, uniform cherries from both control and melatonin treatment were immersed either in melatonin solution (0.5 mM) or in sterile deionized water for 10 min. Thus, experimental fruits were subjected to the following four treatments: Control (spray with dH_2_O and dipping in dH_2_O), melatonin spray = MTS (spray with melatonin and dipping in dH_2_O), melatonin dipping = MTD (spray with dH_2_O and dipping in melatonin), melatonin combination = MTC (spray with melatonin and dipping in melatonin). Following immersion, the fruit were air dried at room temperature for ~30 min. All fruit were cold stored at 0°C and 90% RH for 12 days and then randomly sampled after 8 h at room temperature (20°C) (marked as “post-cold”). At each stage (“harvest” and “post-cold”) and treatment, 5 replicates of 10 fruits without visual defects (represent a biological replicate) were randomly selected for determination of physiological traits. In addition, 3 replicates of 10 fruits including meso- and exo-carp tissues (represent a biological replicate) were immediately snap-frozen in liquid nitrogen and stored at −80°C until use.

### Fruit Ripening Analysis

Color index lightness (L^*^) and Hue angle (*h*°) were measured using a CR-200 Minolta colorimeter (Osaka, Japan) (5). Flesh and dry weight (%) of fruits, total soluble solids (TSS, % Brix), titratable acidity (TA, % malate), and ripening index (TSS/TA) were determined according to Karagiannis (5). The deformation, traction, and penetration forces were measured in 30 fruits following a published protocol (2), using a TA-XT2i Texture Analyzer (Stable Microsystems, Godalming, Surrey, UK). Respiration rate of whole fruits (with stems) was analyzed in five batches of 10 fruits using gas chromatography (Shimadzu GC-2014, Kyoto, Japan) (2). Arithmetic data are presented in Supplementary Table 1.

### UPLC–MS/MS Analysis of Phenolic Compounds

The determination of phenolic compounds was performed as previously described (16). In 100 mg freeze-dried sweet cherry (exo- and meso-carp), 4 mL methanol (80%) were added. Following sonication (20 min), shaking (3 h, 20°C), and after incubation at 4°C (8 h) in the dark, the extract was filtered through a 0.22 μm PFTE membrane into a glass vial. UPLC–MS/MS was carried out on a Waters Acquity system (Milford, MA, USA) consisting of a binary pump, an online vacuum degasser, an autosampler, and a column compartment. The phenolic separation was achieved on a Waters Acquity HSS T3 column 1.8 μm, 100 mm × 2.1 mm at 40°C. The phenolic analysis was performed, as previously described (17). Anthocyanins were quantified as previously described (18) on a RP Acquity UPLC® BEH C18 column (130 Å, 2.1 × 150 mm, 1.7 μm, waters), protected with an Acquity UPLC® BEH C18 pre-column (130 Å, 2.1 × 5 mm, 1.7 μm, waters). Mass spectrometry detection was performed on a Waters Xevo TQMS instrument equipped with an electrospray (ESI) source. Data processing was carried out using the Mass Lynx Target Lynx Application Manager (Waters). Three biological replications were used. The results were expressed as mg 100 g^−1^ FW (Supplementary Table 2).

### Quantitative Real-Time PCR-Based Analysis of Gene Expression

The isolation of mRNA from sweet cherry fruits was conducted in three biological replicates using the RNeasy® Plus Mini Kit from Qiagen (Valencia, CA, USA). Synthesis of cDNA was performed in 10 ng mRNA using the Superscript^TM^ II reverse transcriptase kit (200 U, Life Technologies, Inc.) and a PCR (ProFlex; Thermo Fisher Scientific, Inc.). Quantitative real-time PCR was carried out following the instruction of PowerUp^TM^ SYBR® Green Master Mix (Applied Biosystems, Austin, TX, USA) in a QuantStudio® 5 Real-Time PCR System (96-well, Thermo Fisher Scientific). Primers were designed using Primer3Plus (http://www.bioinformatics.nl/cgi-bin/primer3plus/primer3plus.cgi) and their properties presented in detail at Supplementary Table 3. The qRT-PCR program was performed according to Michailidis (16). Denaturation at 95°C for 10 s, annealing at 54–58°C for 15 s and elongation at 72°C for 20 s. At the end of program melting curves were determined from 65 to 95°C in 0.2°C intervals to validate the formation of expected PCR products. Relative gene expression was analyzed using ΔΔCt method (19) and were expressed as 2^−Δ*ΔCt*^.

### Statistical Analysis

Statistical analysis of all quality traits (Supplementary Table 1), secondary metabolites (Supplementary Table 2) and gene expression was conducted using SPSS (SPSS v21.0, Chicago, USA) by multivariate analysis of variance (MANOVA). Mean values were compared by the *t*-test at harvest or by Duncan's multiple range test, *P* ≤ 0.05 at post-harvest period.

## Results and Discussion

Since melatonin could affect fruit biology (9, 10, 13, 20, 21), studies on fruit response to different time points of melatonin application are important. This study was designed to describe and evaluate physiological and molecular changes caused by exogenous pre- and post-harvest melatonin application as well as by their combination in sweet cherry fruits. The underlying rationale was to provide data relevant to a comprehensive understanding of melatonin-associated sweet cherry on-tree fruit ripening and post-cold responses.

### The Impact of Melatonin in Sweet Cherry Ripening

**Figure 1:**
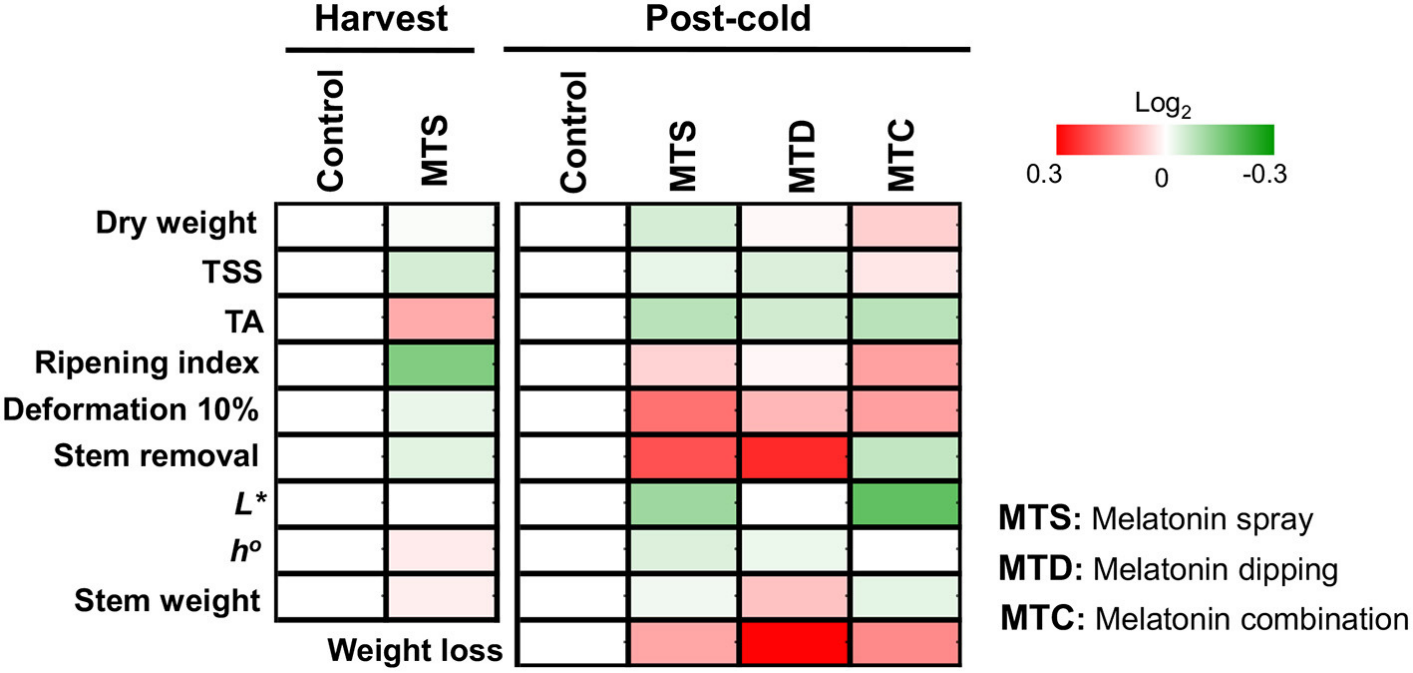
The impact of melatonin pre-harvest foliar spraying and post-harvest fruit dipping, in combination or alone (MTD, melatonin dipping; MTS, melatonin spray; MTC, melatonin combination) in sweet cherry ripening features pictured as heat map profiles. After melatonin pre- and post-harvest application, fruits were cold stored (0°C, RH 90%) for 14 days and subsequently were transferred at ambient temperature (20°C) for 8 h. Fruits were sampled either at the harvest stage (harvest) or following cold storage (post-cold). For each ripening trait, the ratio between treatments to control transformed into log_2_ and depicted with color scale (green indicate relative decrease, red indicate relative increase, and white indicate no fold change). Relative values for each ripening parameter are provided in Supplementary Table 1.

In the present study, we initially characterized the impact of external melatonin on the ripening status of sweet cherry fruits. Data indicated that the combination of pre- and post-harvest application of melatonin (MTC) led to firmer cherries after cold storage (Figure 1 and Supplementary Table 1), implying an effective synergistic role of melatonin sprays and immersion in delaying fruit softening. Fruit firmness in MTC treatment could be linked with a decrease of cell wall degradation during the post-cold period via inhibition of cell-wall-related enzymes such as polygalacturonase, pectin methylesterase, and pectin lyase (22); however, further research is needed to unravel this scenario. Interestingly, the majority of the various tested quality traits such as color, flavor, stem appearance, and weight loss were not influenced by the relative high melatonin level (0.5 mM) applied (Figure 1 and Supplementary Table 1). Tijero (23) reported that low concentrations of melatonin in orchard trees delays sweet cherry ripening in terms of TA content; interestingly, this effect was not observed when applied at higher concentrations. Also, it has been mentioned that low levels (0.1 mM) of melatonin delayed sweet cherry senescence (11, 20). These observations suggest that the anti-ripening effectiveness of melatonin applications will be notably influenced by the concentration applied, among other possible factors.

### Fruit Respiration Exhibited a Reversed Functional in Response to Pre- and Post-harvest Melatonin Feeding

**Figure 2:**
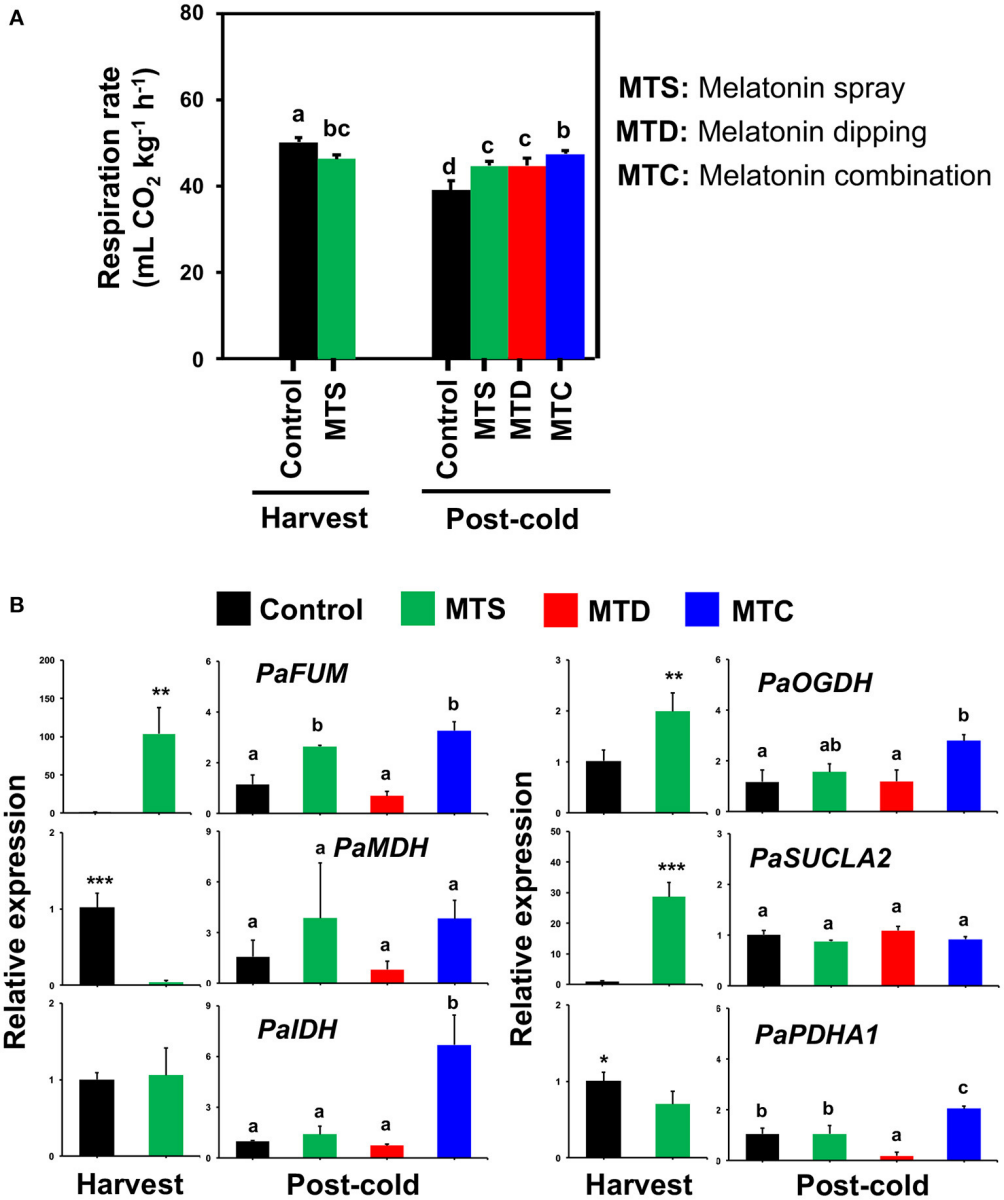
**(A)** Respiration activity of sweet cherries at harvest and post-cold period in response to various pre- and post-harvest melatonin application (MTD, melatonin dipping; MTS, melatonin spray; MTC, melatonin combination). Experiment was performed as described in Figure 1 and under Materials and Methods section. **(B)** Gene expression of fumarase (*PaFUM*), malate dehydrogenase (*PaMDH*), isocitrate dehydrogenase (*PaIDH1*), 2-oxoglutarate dehydrogenase (*PaOGDH*), succinate–CoA ligase [ADP-forming] subunit beta (*PaSUCLA2*) and pyruvate dehydrogenase E1 component subunit beta-3 (*PaPDHA1*) in sweet cherry fruit ripened on-tree (harvest) and under ambient temperature following cold exposure (post-cold). Vertical bars represent SE. Different letters indicate significant differences among treatments according to Duncan's multiple range test; *P* ≤ *0.05*. Mean values with asterisks or different letters indicate significant differences based on Student's *t*-test (*P ≤ 0.05, **P ≤ 0.01, and ***P ≤ 0.001) or Duncan's multiple range test (*P* ≤ 0.05).

Respiration rate was decreased at harvest following pre-harvest melatonin application (MTS) and was then detected to increase following all melatonin treatments in comparison to control at post-cold period, most notably after the combined application (MTC) (Figure 2A), denoting difference of cold-derived melatonin signaling in sweet cherries. Since cold storage is effective for inducing respiration in sweet cherry (5), it is possible that the cold exposure masked the initial respiration-depressed effects of melatonin (Figure 2A). To further study respiration responses, the expression of a set of six genes [*PaFUM*: fumarase, *PaMDH*: malate dehydrogenase, *PaIDH1*: isocitrate dehydrogenase, *PaOGDH*: 2-oxoglutarate dehydrogenase, *PaSUCLA2*: succinate-CoA ligase (ADP-forming) subunit beta, *PaPDHA1*: pyruvate dehydrogenase E1 component subunit beta-3] that are involved in TCA cycle were studied. This analysis indicated that *PaFUM* and *PaOGDH* were induced by MTS and MTC treatments at harvest and post-cold, respectively (Figure 2B, see also Supplementary Table 3). In parallel, an activation of *PaIDH* and *PaPDHA1* was observed at post-cold stage due to MTC treatment (Figure 2B), possibly leading to the increased respiratory activity (Figure 2B). Given that the sweet cherry fruit displayed high respiratory activity and low-temperature storage may induce an uncoupling of the respiratory chain, giving rise to ROS (24), we could assume the existence of an active interplay among respiration, cold stress, and melatonin.

### Melatonin Induced Phenolic Compound Accumulation and Related Genes Expression

**Figure 3:**
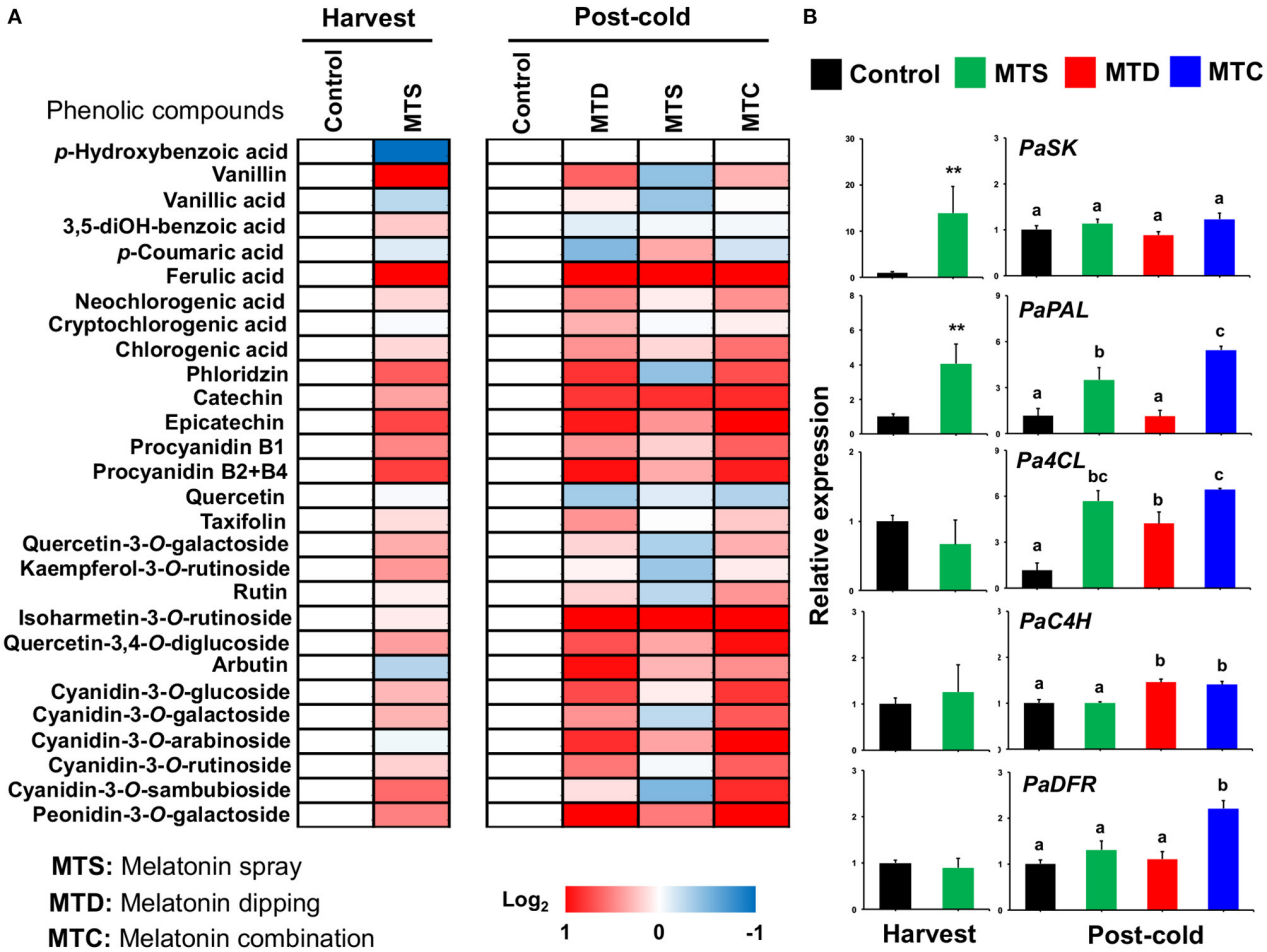
**(A)** Phenolic compounds profile of sweet cherries at harvest and post-cold period in response to various pre- and post-harvest melatonin application (MTD, melatonin dipping; MTS, melatonin spray; MTC, melatonin combination). Experiment was performed as described in Figure 1 and under Materials and Methods section. Each phenolic compound transformed into log_2_ compared to control and depicted with a color scale from blue (relative decrease) to red (relative increase), white indicate no fold change. The quantative results of phenolic compounds are provided in Supplementary Table 3. **(B)** Gene expression of shikimate kinase (*PaSK*), 4-coumarate:CoA ligase 1 (*Pa4CL*), cinnamate-4-hydroxylase (*PaC4H*), phenylalanine ammonia-lyase (*PaPAL*), and dihydroflavonol 4-reductase (*PaDFR*) in sweet cherry fruit ripened on-tree (harvest) and under ambient temperature following cold exposure (post-cold). Vertical bars represent SE. Different letters indicate significant differences among treatments according to Duncan's multiple range test; *P* ≤ 0.05. Mean values with asterisks or different letters indicate significant differences based on Student's *t*-test (*P ≤ 0.05, **P ≤ 0.01, and ***P ≤ 0.001) or Duncan's multiple range test (*P* ≤ 0.05).

Sweet cherries are rich in phenolic acids and flavonoids (mainly anthocyanins), both known for their strong antioxidant activity, and these compounds are primarily responsible for the potential health benefits of cherry uptake (25). Moreover, sweet cherry nutritional quality is strongly dependent on both pre- and post-harvest treatments (16, 26). To obtain information concerning the effect of melatonin on sweet cherry secondary metabolism, analysis of phenolic compounds was performed at harvest and post-cold period. UPLC–MS/MS analysis quantified 28 phenolic compounds in “Ferrovia” cherries that remarkably induced by melatonin at harvest and especially following cold treatment. At harvest, five metabolites (vanillin, ferulic acid, procyanidin B1, procyanidin B2 + B4 and cyanidin-3-*O*-sambubioside) were increased whereas *p*-hydroxybenzoic acid was decreased by melatonin spray (MTS). Interestingly, 11 phenolic compounds, including neochlorogenic acid, chlorogenic acid, phloridzin, epicatechin, procyanidin B1, procyanidin B2+B4, rutin, quercetin-3,4-*O*-diglucoside, cyanidin-3-*O*-glucoside, cyanidin-3-*O*-galactoside, and cyanidin-3-*O*-rutinoside were induced by the combined MTC treatment after cold (Figure 3, see also Supplementary Table 2), signifying that melatonin-induced phenolic accumulation can be sensed differently depending on the pre-cold history of the fruit. The results also suggested that the pre-harvest melatonin application (MTS) enhanced phenol biosynthesis at harvest and after cold storage, but not as effectively as the combination of pre- and post-harvest treatments. A similar increase in the concentration of phenolic compounds after melatonin application was reported recently in table grapes (27). Further, the transcriptional profiles of genes (*PaSK*: shikimate kinase, *Pa4CL*: 4-coumarate: CoA ligase 1, *PaC4H*: cinnamate-4-hydroxylase, *PaPAL*: phenylalanine ammonia-lyase, *PaDFR*: dihydroflavonol 4-reductase) involved in the phenolics biosynthesis was investigated. Results showed that the transcript expression of *PaPAL* was up-regulated by MTS treatment at harvest while the *Pa4CL, PaC4H, PaDFR* were activated by MTC at post-cold (Figure 3), a feature that may explain the observed phenolic compound accumulation, particularly evidenced following cold exposure (Figure 3). Miranda (11) also indicated that post-harvest application of melatonin promoted the expression of sweet cherry genes involved in anthocyanin biosynthesis, lending support to the current results. Altogether, these results documented that melatonin improves sweet cherry nutritional quality by inducing phenolic-associated genes that ultimately lead to increase the content of phenolic compounds.

## Summary

**Figure 4:**
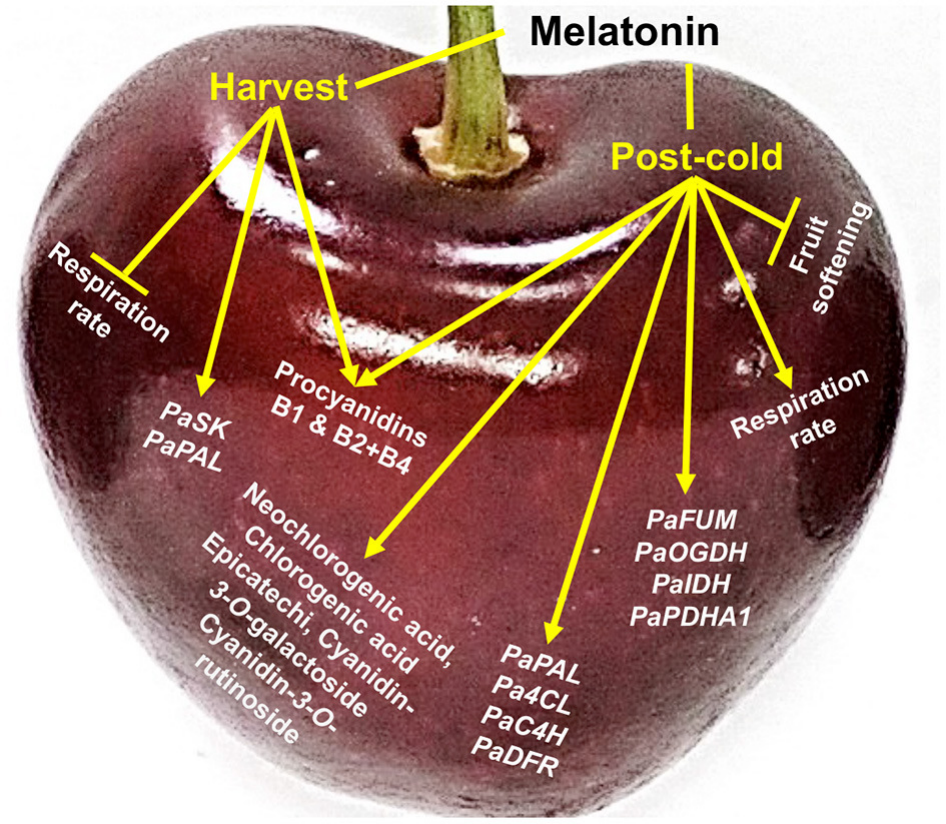
Schematic presentation of the impact of melatonin in sweet cherries (cv. Ferrovia) at harvest stage (harvest) and following cold storage (post-cold).

This article reports major ripening and post-cold events that occur due to external pre- and post-harvest application of melatonin in sweet cherry. On-tree fruit respiration was depressed by melatonin pre-harvest spray while the combination of the two melatonin treatments delayed postharvest fruit softening. The current results also highlight that sweet cherry response to melatonin involves a cold-dependent activation of the respiration and up-regulation of TCA cycle genes. Melatonin application induced phenolic compound accumulation, including proanthocyanidins and anthocyanins, through the up-regulation of various related genes (e.g., *PaPAL, Pa4CL, PaC4H*, and *PaDFR*) at harvest and especially at post-cold period (Figure 4). The data reported here, in combination with recent findings, will expand our understanding of melatonin-responsive ripening changes in sweet cherry and facilitate our ability to predict how fruit will respond to melatonin application.

## Data Availability Statement

The raw data supporting the conclusions of this article will be made available by the authors, without undue reservation.

## Author Contributions

MM and AM designed and conceived the research and drafted the manuscript. MM, GT, ES, EK, IG, and SM analyzed the data and interpreted the results. All authors contributed to the article and approved the submitted version.

## Conflict of Interest

The authors declare that the research was conducted in the absence of any commercial or financial relationships that could be construed as a potential conflict of interest.
