# Supplementary material for: Pre- and Post-harvest Melatonin Application Boosted Phenolic Compounds Accumulation and Altered Respiratory Characters in Sweet Cherry Fruit
Source: Front Nutr. 2021 Jun 9;8:695061. doi: 10.3389/fnut.2021.695061 (PMC8219925; doi:10.3389/fnut.2021.695061)
Supplement: Supplementary file 1 [file Data_Sheet_1.PDF]

Supplementary Material

Supplementary Table S1. Ripening traits of fruits at harvest and during postharvest period.

Supplementary Table 1. Physiolocal traits of fruits at harvest and during post-cold period.

		Dry weight (%)			TSS (%, Brix)			TA (%, malate)			Ripening index (RI)			Deformation 10% (Nmm^{-1})		
		Mean	SD	Duncan	Mean	SD	Duncan	Mean	SD	Duncan	Mean	SD	Duncan	Mean	SD	Duncan
Harvest	Control	17.6	0.76	a	18.36	0.11	bc	1.12	0.06	a	16.39	0.95	b	0.59	0.04	a
	MTS	17.51	0.36	a	17.76	0.58	c	1.2	0.06	a	14.8	0.6	b	0.58	0.04	a
12 days at 0°C	Control	17.7	0.32	a	19.46	0.95	ab	1.1	0.04	a	17.69	0.34	a	0.49	0.05	c
	MTS	17.12	0.31	a	19.1	0.1	ab	1.04	0.02	a	18.36	0.26	a	0.55	0.04	ab
	MTD	17.79	0.59	a	18.9	0.45	abc	1.06	0.05	a	17.83	0.52	a	0.52	0.05	bc
	MTC	18.42	0.5	a	19.86	0.85	a	1.04	0.03	a	19.1	0.25	a	0.53	0.04	ab

		Stem removal (N)			Color index *L**			Color index *h°*			Stem weight (%)			Weight loss (%)		
		Mean	SD	Duncan	Mean	SD	Duncan	Mean	SD	Duncan	Mean	SD	Duncan	Mean	SD	Duncan
Harvest	Control	2.2	0.64	a	22.66	0.57	a	19	1	a	1.39	0.01	a	-	-	
	MTS	2.15	0.52	a	22.66	1.15	a	19.3	1.15	a	1.41	0.02	a	-	-	
12 days at 0°C	Control	2.09	1.33	a	22.33	1.52	a	21.6	0.57	a	0.97	0.02	b	2.5	0.37	a
	MTS	2.41	0.92	a	20.66	3.05	a	21	1	a	0.96	0.02	b	2.69	0.25	a
	MTD	2.49	1.13	a	22.33	1.52	a	21.3	2.08	a	1.02	0.07	b	3.07	0.14	a
	MTC	1.99	0.85	a	19.66	2.88	a	21.6	1.52	a	0.95	0.03	b	2.75	0.05	a

***MTS** Melatonin spray treatment*
***MTD** Melatonin dipping treatment*
***MTC** Melatonin combination treatment*
***P-value** ≤ 0.05*
Different letters indicate significant difference based on Duncan's multiple range test

Supplementary Table S2. Quantitative results of polyphenolic compounds.

Supplementary Table S2. Quantative results of polyphenolic compounds

		Harvest						12 days at 0 °C											
		Control			MTS			Control			MTS			MTD			MTC		
A/A	Phenolic compounds	Mean		SD	Mean		SD	Mean		SD	Mean		SD	Mean		SD	Mean		SD
1	*p*-Hydroxybenzoic acid	0.28	*	0.099	ND			ND			ND			ND			ND		
2	Vanillin	ND			0.123	*	0.021	0.084		0.022	0.127		0.045	0.062		0.023	0.104		0.078
3	Vanillic acid	0.06		0.048	0.05		0.029	0.065		0.047	0.068		0.02	0.049		0.008	0.064		0.043
4	3,5-diOH-benzoic acid	0.075		0.031	0.087		0.023	0.087		0.019	0.08		0.016	0.084		0.017	0.084		0.016
5	*p*-Coumaric acid	0.43		0.062	0.393		0.049	0.414		0.083	0.296		0.145	0.524		0.197	0.367		0.085
6	Ferulic acid	ND			0.116	*	0.068	0.001		0	0.091		0.003	0.066		0.045	0.06		0.051
7	Neochlorogenic acid	50.92		3.19	56.66		2.84	49.06	b	7.95	66.46	a	4.49	51.59	b	5.93	66.27	a	3.46
8	Cryptochlorogenic acid	2.11		0.398	2.08		0.389	1.84		0.665	2.28		0.197	1.81		0.318	1.93		0.097
9	Chlorogenic acid	5.31		0.407	5.91		0.66	5.18	c	0.955	6.98	ab	1.04	5.78	bc	0.053	7.63	a	0.474
10	Phloridzin	0.04		0.024	0.069		0.033	0.073	b	0.006	0.127	a	0.002	0.054	b	0.04	0.118	a	0.024
11	Catechin	10.97		3.66	14.11		3.59	7.2		6.24	12.48		0.61	12.7		3.01	12.87		1.28
12	Epicatechin	2.41		0.835	4		0.897	4.03	b	1.03	7.53	a	0.603	5.38	b	1.07	8.42	a	0.477
13	Procyanidin B1	0.898		0.092	1.24	*	0.154	1.33	c	0.17	1.77	ab	0.117	1.51	bc	0.316	2.05	a	0.116
14	Procyanidin B2 + B4	1.98		0.394	3.35	*	0.689	3.75	b	0.346	7.19	a	0.91	4.72	ab	1.47	6.97	a	2.1
15	Quercetin	0.08		0.004	0.08		0.001	0.079		0.021	0.062		0.053	0.073		0.006	0.064		0.055
16	Taxifolin	0.145		0.02	0.16		0.028	0.143	b	0.009	0.192	a	0.007	0.142	b	0.018	0.166	ab	0.019
17	Quercetin-3-*O*-galactoside	4.22		0.984	5.3		0.759	5.25	ab	1.1	5.89	a	0.104	4.21	b	0.506	6.52	a	0.437
18	Kaempferol-3-*O*-rutinoside	1.18		0.331	1.57		0.598	1.45		0.323	1.5		0.214	1.1		0.246	1.53		0.353
19	Rutin	7.4		0.955	7.72		0.918	7.76	bc	1.69	8.75	ab	0.426	6.46	c	1.08	10.36	a	0.707
20	Isorhamnetin-3-*O*-rutinoside	0.378		0.061	0.4		0.019	0.129		0.222	0.398		0.069	0.354		0.037	0.404		0.042
21	Quercetin-3,4-*O*-diglucoside	0.106		0.026	0.139		0.053	0.106	c	0.03	0.17	ab	0.053	0.135	bc	0.003	0.204	a	0.003
22	Arbutin	0.097		0.023	0.08		0.013	0.059		0.013	0.114		0.019	0.072		0.034	0.08		0.019
23	Cyanidin-3-*O*-glucoside	1.79		0.128	2,177		0.334	2.27	b	0.299	3.72	a	0.54	2.39	b	0.751	3.94	a	0.465
24	Cyanidin-3-*O*-galactoside	50.83		6.53	62.35		13.63	93.6	b	16.41	125.99	a	11.6	78.59	b	21.77	146.73	a	16.92
25	Cyanidin-3-*O*-arabinoside	0.047		0.012	0.045		0.013	0.043		0.003	0.076		0.02	0.055		0.015	0.098		0.033
26	Cyanidin-3-*O*-rutinoside	137.24		11.83	155.82		24.46	164.53	b	17,394	238.17	a	25.05	160.25	b	41.48	253.53	a	24.08
27	Cyanidin-3-*O*-sambubioside	0.02		0.002	0.03	**	0.001	0.029		0.01	0.031		0.004	0.02		0.008	0.052		0.024
28	Peonidin-3-*O*-galactoside	0.014		0.007	0.02		0.092	0.013		0.014	0.036		0.017	0.019		0.009	0.035		0.014

ND: no detected

All compounds expressed as mg $100g^{-1}$

Different letter indicate significant difference during postharvest period based on Duncan's multiple range test, P ≤ 0.05

*** P ≤ 0.05**

**** P ≤ 0.01**

Supplementary Table S3. Transcripts properties and primer sequence.

Supplementary Table 3. Transcripts properties and primer sequence.									
				Primer Sequence					
Housekeeping Transcript				Forward	Tm	Reverse	Tm	Abbreviations	Accesion number
http://dx.doi.org/10.1016/j.scienta.2014.10.027		Actin 7 (ACT7)/actin 2		CTTGCATCCCTCAGCACCTT	62.1	TCCTGTGGACAATGGATGGA	62.1	*ACT*	***EC969944***
**Transcripts related to TCA cycle**									
				Primer Sequence					
Substrate metabolite	Product metabolite	Enzyme	EC number	Forward	Tm	Reverse	Tm	Abbreviations	Accesion number
fumarate	malate	fumarase	4.2.1.2	CCGCATGTATCAGCTTGCAC	60.5	TCGGCGGTGACAAATGGTAA	58.4	*PaFUM*	*XM_021949489.1*
malate	oxaloacetate	malate dehydrogenase	1.1.1.37	GAACCTGCAGCTGAGTCCTT	60.5	TTCCGTGGGAATCCACCAAC	60.5	*PaMDH*	*XM_021948632.1*
Isocitrate	Oxalosuccinate	isocitrate dehydrogenase	1.1.1.42	ATCTTTGCTTGGTCCCGAGG	60.5	AGTCGAAACACAGGCTGCTT	58.4	*PaIDH1*	*XM_021946692.1*
Oxalosuccinate	2-oxoglutarate	isocitrate dehydrogenase	1.1.1.42	ATCTTTGCTTGGTCCCGAGG	60.5	AGTCGAAACACAGGCTGCTT	58.4	*PaIDH1*	*XM_021946692.1*
2-oxoglutarate	3-Carboxy-1-hydroxypropyl-ThPP	2-oxoglutarate dehydrogenase, mitochondrial	1.2.4.2	GGAATGTCTCACAGAGGAAGACT	62.9	CCAGTCCCTGTGTATAGTCCAA	62.1	*PaOGDH*	*XM_021958087.1*
3-Carboxy-1-hydroxypropyl-ThPP	S-Succinyldihydrolipoamide-E	2-oxoglutarate dehydrogenase, mitochondrial	1.2.4.2	GGAATGTCTCACAGAGGAAGACT	62.9	CCAGTCCCTGTGTATAGTCCAA	62.1	*PaOGDH*	*XM_021958087.1*
succinyl-CoA	succinate	succinate--CoA ligase [ADP-forming] subunit beta, mitochondrial	6.2.1.4	GCAAACAGGTCCTCAGGGAA	60.5	CCAGCAGTTTTGCGATCCAG	60.5	*PaSUCLA2*	*XM_021959105.1*
Puryvate	2-Hydroxyethyl-ThPP	pyruvate dehydrogenase E1 component subunit beta-3, chloroplastic	1.2.4.1	CGTCCTCTGGACAGGAACAC	62.5	CACCATGCTGAGGAAACCCT	60.5	*PaPDHA1*	*XM_021956141.1*
2-Hydroxyethyl-ThPP	S-Acetyldihydrolipoamide-E	pyruvate dehydrogenase E1 component subunit beta-3, chloroplastic	1.2.4.1	CGTCCTCTGGACAGGAACAC	62.5	CACCATGCTGAGGAAACCCT	60.5	*PaPDHA1*	*XM_021956141.1*
**Transcripts of secondary metabolism**									
				Primer Sequence					
Substrate metabolite	Product metabolite	Enzyme	EC number	Forward		Reverse		Abbreviations	Accesion number
Shikimate	Shikimate 5-phosphate	shikimate kinase	2.7.1.71	GAACTGGTTCTCGCCCTCTT	60.5	TCAAAAAGGCTTGTCAGACGC	59.5	*PaSK*	*XM_021946841.1*
trans-Cinnamate	Cinnamoyl-CoA	4-coumarate:CoA ligase 1	6.2.1.12	GGGGTTGATCGAGAAGCACA	60.5	CGTACTTGTCGAGATCGGGG	62.5	*Pa4CL*	*XM_021946313.1*
p-Coumaric acid	p-Coumaroyl-CoA	4-coumarate:CoA ligase 1	6.2.1.12	GGGGTTGATCGAGAAGCACA	60.5	CGTACTTGTCGAGATCGGGG	62.5	*Pa4CL*	*XM_021946313.1*
trans-Cinnamate	p-Coumaric acid	cinnamate-4-hydroxylase	1.14.13.11	TGACCGTCCCTTTCTTCACG	60.5	GGTGCTTCTTCACGTCCTCA	60.5	*PaC4H*	*GU990522.1*
Cinnamoyl-CoA	p-Coumaroyl-CoA	cinnamate-4-hydroxylase	1.14.13.11	TGACCGTCCCTTTCTTCACG	60.5	GGTGCTTCTTCACGTCCTCA	60.5	*PaC4H*	*GU990522.1*
L-Phenylalanine	trans-Cinnamate	phenylalanine ammonia-lyase	4.3.1.24	TGGGCTCAAAGGTGCTGAAA	60	TGGACATGGTTGGTGACAGG	60	*PaPAL*	*XM_021971014.1*
Dihydroquercetin	Leucocyanidin	bifunctional dihydroflavonol 4-reductase/flavanone 4-reductase	1.1.1.219	AGACAGTTCGAAGGCTGGTG	60	ATCGCTCCAGTTGGTTTCGT	60	*PaDFR*	*XM_021975874.1*
